# Impact of academic self-efficacy on online learning outcomes: a recent literature review

**DOI:** 10.17179/excli2024-7502

**Published:** 2024-07-13

**Authors:** Satoru Yokoyama

**Affiliations:** 1 Saitama University, 255 Shimookubo, Sakura Ward, Saitama, 338-8570, Japan

**Keywords:** academic self-efficacy, academic performance, online learning, e-learning, distance learning

## Abstract

In recent years, the concept of self-efficacy has garnered attention in educational psychology research on motivation. Within an academic context, academic self-efficacy (ASE) reflects learners' belief in their ability to achieve educational goals. However, most research has focused on traditional face-to-face classroom settings, with little exploration in distance learning environments like online and e-learning. The current review aims to update a previous study (Yokoyama, 2019[40]) and examine differences in online learning types: asynchronous, synchronous, and blended learning. The study's findings reveal that in mixed environments combining synchronous and asynchronous elements, or in blended settings merging face-to-face classes with asynchronous learning, ASE positively impacts academic performance akin to traditional face-to-face classes. However, in asynchronous online learning environments, ASE's influence on academic performance might be slightly weaker compared to synchronous learning environments. The paper will subsequently discuss the pedagogical implications derived from these results.

## Introduction

In recent years, the concept of self-efficacy has garnered attention in research on motivation in educational psychology. Self-efficacy (SE), as developed by Bandura (1997[4]), refers to a belief in one's ability to organize and carry out a series of actions necessary to achieve a given outcome. Self-efficacy entails a belief regarding an individual's ability to organize and execute the courses of action necessary to achieve desired performance. The influence of SE has been explored across various psychological disciplines in areas such as smoking cessation, dietary behavior change, and addiction relapse (Povey et al., 2000[28]). This SE is a fundamental aspect of human agency believed to be positively associated with academic success (Bandura, 1977[5], 1997[4], 2012[6]; Pajares and Schunk, 2001[27]). Within an academic context, SE is often conceptualized as academic self-efficacy (ASE), defining learners' belief about their ability to successfully achieve educational goals (Elias and MacDonald, 2007[14]). Extensive research has examined the notion that ASE positively motivates academic performance (Honicke and Broadbent, 2016[17]; Vancouver et al., 2001[36]). For instance, heightened ASE may directly correlate with students' coping behaviors. Students with elevated ASE are more inclined to persist in their endeavors and demonstrate resilience in the face of challenges (Adams et al., 2020[1]; Musa, 2020[24]). Several meta-analytic studies have consistently found a positive correlation between ASE and academic achievement, regardless of the educational context in which ASE is assessed (Honicke and Broadbent, 2016[17]; Multon et al., 1991[23]; Richardson et al., 2012[29]; Robbins et al., 2004[30]).

ASE is linked to students' selection of learning activities, level of exertion, and perseverance in overcoming obstacles (Bandura, 1986[7]; Bong and Skaalvik, 2003[8]; Pajares, 1996[26]; Schunk, 1991[32]). Furthermore, research suggests that while learners with high academic performance tend to exhibit higher ASE, there is also evidence that elevated ASE enhances academic performance (Talsma et al., 2018[35], for a review). This implies that interventions aimed at increasing ASE could bolster motivation for learning and consequently enhance academic performance.

Regarding the learning environment, aside from traditional face-to-face classroom settings, learners also engage in distance learning modalities such as e-learning. Theoretically, two primary types of distance learning exist: synchronous (synchro) and asynchronous (asynchro, Gunawardena and McIsaac, 2004[16]). In synchro learning, individuals collaborate in groups simultaneously via the Internet or other platforms. Synchro learning enables educators and learners from disparate locations to engage in an environment akin to face-to-face classroom instruction. In contrast, asynchro learning involves learners completing tasks independently and at their own pace. Asynchro learning facilitates distance education without temporal constraints, allowing learners to study at their convenience. Thus, while synchro learning mirrors traditional classroom education, the asynchro one presents distinct characteristics in the learning environment. Additionally, blended learning-a method combining multiple approaches-often integrates traditional face-to-face classes with asynchro distance learning as an extracurricular learning platform. Consequently, ASE may influence academic performance differently due to the varying characteristics of distance learning settings. 

However, while the majority of research has concentrated on traditional face-to-face classroom education settings (e.g., Honicke and Broadbent, 2016[17]; Richardson et al., 2012[29]; Robbins et al., 2004[30]; Talsma et al., 2018[35]), there has been minimal exploration in distance learning environments such as online learning and e-learning (Yokoyama, 2019[40]). To my knowledge, Yokoyama (2019[40]) was the sole review examining the impact of ASE on academic performance in online learning settings, published in 2019 with a research scope extending up to 2018. However, this review did not account for the differences in online learning types, such as synchro, asynchro, and blended learning. Therefore, the aim of the current review is to identify previous studies investigating the correlation between ASE and academic performance in online learning settings, particularly from 2018 to 2023, using the same methodology as Yokoyama (2019[40]), and supplementing Yokoyama's findings. Subsequently, the current study comprehensively evaluates all listed studies, considering the distinctions among online learning types. 

## Methods

This study utilized the search method outlined in Yokoyama (2019[40]). Initially, the Web of Science served as the primary database, with Yokoyama (2019[40]) being based on search results as of June 2018. Following this precedent, the current study employed the same search parameters to explore papers published from 2018 to 2023 (conducted in January 2024). The search query was constructed as follows: TS = [(“academic self-efficacy” OR “academic self-efficacy”) AND (“academic performance” OR “academic performance” OR “academic performance” or “learning outcomes”) AND (e-learning OR “distance learning” or online learning” or “distance education”)]. Only papers written in English that investigated the correlation or modeling of ASE and academic performance in online learning settings were manually extracted. The found studies in Yokoyama (2019[40]) were then integrated into the current study's results to cover studies published before 2018. Subsequently, all integrated studies were reviewed, with new information about synchro, asynchro or blended settings added for each study. 

## Results

The initial search yielded 15 papers. One paper was excluded due to its non-English language (González-Benito et al., 2021[15]). Additionally, six papers were excluded as they did not utilize ASE scores or measure academic performance (Adeshola and Agoyi, 2023[2]; Oztürk et al., 2022[25]; Rohmani and Andriani, 2021[31]; Wang et al., 2023[37]; Wong et al., 2023[39]; Zhang et al., 2023[42]). Consequently, eight papers remained in the current study's search (Aldhahi et al., 2021[3]; Broadbent and Howe, 2023[9]; Cheng et al., 2023[10]; Daşcı et al., 2023[13]; Huang and Wang, 2023[18]; Sutherland et al., 2023[33]; Talsma et al., 2023[34]; Won et al., 2023[38]). These results, in conjunction with those from Yokoyama 2019[40] (Cho and Shen, 2013[11]; Crippen et al., 2009[12]; Lynch and Dembo, 2004[22]; Kitsantas and Chow, 2007[21]; Yukselturk and Bulut, 2007[41]), are summarized in Table 1(Tab. 1) (References in Table 1: Aldhahi et al., 2021[3]; Broadbent and Howe, 2023[9]; Cheng et al., 2023[10]; Cho and Shen, 2013[11]; Crippen et al., 2009[12]; Daşcı et al., 2023[13]; Huang and Wang, 2023[18]; Joo et al., 2013[19]; Kitsantas and Chow, 2007[21]; Lynch and Dembo, 2004[22]; Sutherland et al., 2023[33]; Talsma et al., 2023[34]; Won et al., 2023[38]; Yokoyama, 2019[40]; Yukselturk and Bulut, 2007[41]). 

In terms of statistical outcomes, while Yokoyama (2019[40]) showed significant positive correlations between ASE and academic performance in 4 out of 6 studies, the current review identified such correlations in only 7 out of 8 studies. Overall, while three studies indicated no significant correlations, eleven studies demonstrated statistically significant positive correlations.

Regarding online learning settings, no synchro settings were encountered. Eight studies focused on asynchro settings, while three explored mixed or blended settings. However, unfortunately, three previous studies did not clearly specify the type of settings investigated. Among studies focusing on asynchro settings, five out of eight (three out of five in Yokoyama (2019[40]), and two out of three in the current review) revealed statistically significant positive correlations between ASE and academic performance, while three (two in Yokoyama (2019[40]) and one in the current review) did not. In mixed or blended settings, all three studies demonstrated statistically significant positive correlations. 

## Discussion

This literature review explored the effectiveness of ASE on learning outcomes in online learning environments. Specifically, this review provided updated findings since the 2019 review paper, incorporating various types of online learning environments-synchro, asynchro, and blended-which were not previously addressed. Newly discovered papers were added to those listed in the 2019 review. Consequently, while three studies showed no significant correlations, eleven studies demonstrated statistically significant positive correlations between ASE and academic performance. 

Among the previous studies investigating asynchro learning environments, it is important to discuss why some studies found no correlations between ASE and academic performance. One possible interpretation could be methodological differences among the studies, including the countries or regions where the research was conducted, the type of participants, the study sample size, and the type of data used.

Firstly, regarding the countries and regions where the research was conducted, the papers that showed no correlation hailed from China, the United States, and Australia. Conversely, papers that indicated a correlation included those from the United States and Australia, but not China. In Turkey, both papers reported significant correlations in the asynchro environment. While there may be influences from the differing countries or regions, the limited number of studies makes it challenging to draw definitive conclusions at this stage.

Secondly, all studies listed in the current review involved college students as participants. Therefore, the type of participants is unlikely to have influenced the reported correlation results.

Thirdly, the study sample size is also unlikely to have significantly impacted the asynchro correlation results. For instance, the sample sizes of papers reporting significant correlations in the asynchro environment ranged from 80 to 897. Conversely, the sample sizes of papers reporting no correlations ranged from 64 to 174. Although there was a slight discrepancy in sample sizes, it is inconclusive to suggest that results clearly differ based on sample sizes.

Fourthly, the type of data used is also improbable to have significantly influenced the asynchro correlation results. Specifically, the ASE scale used in papers reporting correlations in an asynchro environment included the Motivated Strategies for Learning Questionnaire (MSLQ), an author-created scale, and the scale developed in Kandemir (2014[20]). Conversely, the ASE scales used in papers reporting no correlation included the MSLQ and author-created scales. In terms of measuring academic achievement, both sets of papers used course grades and GPA. 

There are two other possible explanations than the methodological differences among the studies. One is the possibility that differences in learning between synchro and asynchro environments may be influencing the results. Another possibility, which is often not mentioned in the papers, may be the distribution of ASE and academic performance data.

The former suggests that differences in learning between synchro and asynchro environments may influence the results. In mixed or blended environments that combine face-to-face and online classes, ASE positively impacts academic performance, similar to traditional face-to-face classes. Conversely, in asynchro settings, where students study independently without direct communication, maintaining motivation for learning might be challenging due to the absence of synchro learning characteristics that facilitate communication. This interpretation aligns with social cognitive theory, which emphasizes the role of communication and encouragement in enhancing SE (Bandura, 1997[4]).

The latter explanation concerns the distribution of ASE and academic performance data. In studies examining correlations, if the data is not normally distributed and exhibits a biased distribution leading to a ceiling effect, the test results may be inaccurate. Although the current study could not confirm this in all previous studies that found no correlation, depending on the university or class, there might be bias in ASE, with some students scoring high overall while others score low. Similarly, grades might be biased upward or downward based on the university level and class difficulty. Thus, it is crucial for future researchers to clearly report the distribution of ASE and academic performance data. 

## Pedagogical Implications

The findings of this study suggest that in mixed environments combining synchro and asynchro learning, or in blended environments merging face-to-face and online classes, ASE significantly boosts academic performance, akin to traditional face-to-face classes. Conversely, in asynchro online learning, the impact of ASE on academic performance may be slightly weaker due to the absence of synchro elements for communication. Therefore, it is advisable that in asynchro environments, incorporating means for teacher-student communication and integrating synchro elements could enhance ASE's impact, potentially matching the strong influence observed in traditional face-to-face classes. 

## Declaration

### Acknowledgments

This work was supported by JSPS KAKENHI Grant Number JP22K00811. The author would like to thank Editage (www.editage.jp) for English language editing. 

### Conflict of interest

The author declares that he has no conflict of interest. 

## Figures and Tables

**Table 1:**
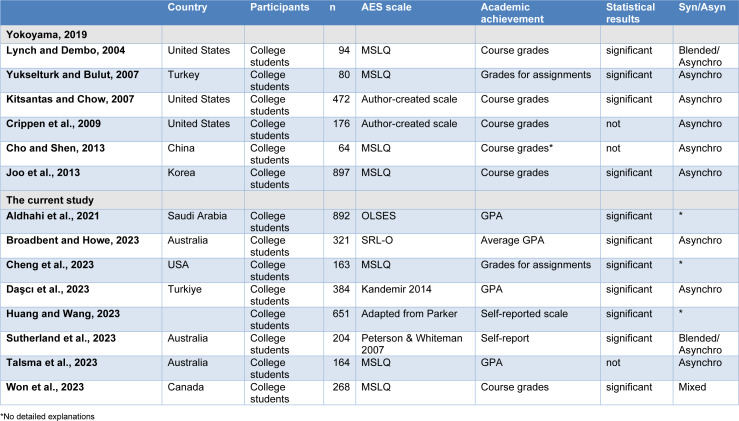
Summary of included studies
